# Semantic word category processing in semantic dementia and posterior cortical atrophy

**DOI:** 10.1016/j.cortex.2017.04.016

**Published:** 2017-08

**Authors:** Zubaida Shebani, Karalyn Patterson, Peter J. Nestor, Lara Z. Diaz-de-Grenu, Kate Dawson, Friedemann Pulvermüller

**Affiliations:** a Medical Research Council, Cognition and Brain Sciences Unit, Cambridge, UK; b Linguistics Department, College of Humanities and Social Sciences, United Arab Emirates University, United Arab Emirates; c Department of Clinical Neurosciences, University of Cambridge, UK; d German Center for Neurodegenerative Diseases (DZNE), Magdeburg, Germany; e Tecnalia Research and Innovation Center, Health Division, Neurotechnology Unit, Bizkaia Technology Park, Derio, Spain; f Brain Language Laboratory, Department of Philosophy and Humanities, WE4, Freie Universität Berlin, Berlin, Germany; g Berlin School of Mind and Brain, Humboldt Universität zu Berlin, Berlin, Germany; h Einstein Center for Neurosciences, Berlin, Germany

**Keywords:** Semantic Dementia, Posterior Cortical Atrophy, Category specificity, Word processing, Semantics

## Abstract

There is general agreement that perisylvian language cortex plays a major role in lexical and semantic processing; but the contribution of additional, more widespread, brain areas in the processing of different semantic word categories remains controversial. We investigated word processing in two groups of patients whose neurodegenerative diseases preferentially affect specific parts of the brain, to determine whether their performance would vary as a function of semantic categories proposed to recruit those brain regions. Cohorts with (i) Semantic Dementia (SD), who have anterior temporal-lobe atrophy, and (ii) Posterior Cortical Atrophy (PCA), who have predominantly parieto-occipital atrophy, performed a lexical decision test on words from five different lexico-semantic categories: colour (e.g., *yellow*), form (*oval*), number (*seven*), spatial prepositions (*under*) and function words (*also*). Sets of pseudo-word foils matched the target words in length and bi-/tri-gram frequency. Word-frequency was matched between the two visual word categories (colour and form) and across the three other categories (number, prepositions, and function words). Age-matched healthy individuals served as controls. Although broad word processing deficits were apparent in both patient groups, the deficit was strongest for colour words in SD and for spatial prepositions in PCA. The patterns of performance on the lexical decision task demonstrate (a) general lexicosemantic processing deficits in both groups, though more prominent in SD than in PCA, and (b) differential involvement of anterior-temporal and posterior-parietal cortex in the processing of specific semantic categories of words.

## 1 Introduction

The brain regions involved in language processing seem to extend well beyond the classical Broca (inferior frontal) and Wernicke (superior temporal) areas of the dominant hemisphere. Furthermore, evidence from functional neuroimaging indicates that, in addition to regions responsive to words in general, different brain regions are engaged during the processing of various word classes, with activated areas depending in part on the type of meaning. For example, words pertaining to colours and colour knowledge have been found to activate regions of the temporal lobe anterior to colour perception areas in posterior temporal cortex (Martin et al., 1995, Simmons et al., 2007), whereas words related to object shape engage more posterior-temporal regions (Moscoso Del Prado Martin et al., 2006, Pulvermüller and Hauk, 2006). Even more specifically, words referring to actions typically performed using the face (e.g., *chew*), arm (*clap*) and leg (*jump*) have been shown to activate the very same areas in the motor and premotor cortex that control movement of those specific body parts (Grisoni et al., 2016, Hauk et al., 2004, Hauk et al., 2008, Kemmerer, 2015, Shtyrov et al., 2014, Shtyrov et al., 2004). Activation of sensori-specific brain regions in semantic processing has been documented for odour-related words such as *jasmine* (Gonzalez et al., 2006), words semantically related to sounds such as *telephone* (Kiefer, Sim, Herrnberger, Grothe, & Hoenig, 2008), and taste-related words such as *salt* (Barros-Loscertales et al., 2012). These and similar results showing category-specific activation patterns have led to the suggestion that different sets of cortical areas contribute differentially to conceptual semantic processing (for review see Pulvermüller, 1999, Pulvermüller, 2013).

If language processing relies on distributed semantic circuits that extend into sensory and motor regions, then lesions in and close to these modality-preferential areas should have differential effects on the processing of different word categories. Indeed, over the past three decades, numerous patient studies have reported such category-specific impairments. Double dissociations have been found in the processing of animals versus tools and living versus non-living things (e.g., Gainotti, 2004, Warrington and McCarthy, 1983, Warrington and McCarthy, 1987, Warrington and Shallice, 1984). Investigations of noun and verb processing have reported that the two word categories are differentially affected following lesions due to stroke or neurodegenerative disease (Bak et al., 2001, Boulenger et al., 2008, Cotelli et al., 2006, Damasio and Tranel, 1993, Kemmerer et al., 2012, Neininger and Pulvermüller, 2003). For instance, patients with motor neuron disease (MND), a neurodegenerative condition predominantly affecting the sensorimotor system, were consistently more impaired on action verbs than object nouns in picture naming and comprehension tasks (Bak et al., 2001); and patients with Parkinson's disease, a condition primarily characterised by motor disorders, had deficits in processing action verbs compared to concrete nouns (Boulenger et al., 2008).

The conclusions of these imaging and neuropsychological studies have not, however, been universally endorsed. For example, since object nouns and action verbs differ not only in semantic but also in lexical and morpho-syntactic characteristics (Schnur et al., 2009, Vigliocco et al., 2011), differences in processing the two word categories may not provide clear evidence for category-specific semantic networks (Bird et al., 2000, Mahon and Caramazza, 2008). Furthermore, some studies have not controlled for critical stimulus variables such as word length, frequency, imageability or orthographic/phonological patterns. For example, because many nouns are object names, they tend to be more imageable and visually-related than verbs. Verbs, on the other hand, typically have higher word stem frequency than nouns. Also, nouns and verbs could in theory activate different cortical areas due to their phonological makeup (see de Zubicaray, Arciuli, & McMahon, 2013 for an analysis of the latter in this context), given that phonological features seem to relate to different cortical loci (Evans and Davis, 2015, Pulvermüller et al., 2006, Schomers et al., 2015). Controlling for such psycholinguistic stimulus variables is vital since syntactic, lexical and semantic features, and even aspects of surface structure, all contribute to distinctions between nouns and verbs (Bird et al., 2000, Funnell and Sheridan, 1992, Pulvermüller, 1999).

When such psycholinguistic variables were tightly controlled in imaging studies, the specific activation of modality preferential areas during language processing was confirmed for well-matched semantic symbol types, such as words related to colour and form or the body-part specific subtypes of action words (Grisoni et al., 2016, Kiefer and Pulvermüller, 2012). However, the activation of an area during symbol processing does not uniquely identify the role of these activated regions in semantic processing. Even unambiguous functional magnetic resonance imaging (fMRI) results do not establish a necessary role of action-perception systems for semantic processing. Differential deficits across categories associated with specific lesion sites may, at least in some researchers' views, constitute more compelling evidence for the necessity of such contributions to semantic processing. The current study assessed semantic word category processing in patients from two different clinical groups: (i) Semantic Dementia (SD), the temporal-lobe variant of Frontotemporal Dementia, which primarily affects anterior-temporal regions, and (ii) Posterior Cortical Atrophy (PCA), an atypical variant of Alzheimer's disease (AD), which predominantly affects posterior parieto-occipital regions (a description of each syndrome is provided below). These groups were selected for the study because they have relatively well-defined, if not focal, bilateral lesions in areas outside perisylvian language cortex. Areas affected in each condition are proposed to make a category-specific contribution to semantic word processing: the anterior temporal lobe to the processing of words related to colour and object knowledge; the parietal cortex to the processing of words related to spatial information (Damasio et al., 2001, Noordzij et al., 2008, Tranel and Kemmerer, 2004). The study of word processing in these patients whose degenerative conditions affect specific regions of the brain while leaving other regions—especially ‘core’ language areas—relatively spared may therefore provide valuable information on the organisation of language and semantic knowledge in the brain. A lexical decision experiment using printed words as stimuli was designed to address hypotheses about category-specific semantic deficits in each patient group.

**Fig. 1:**
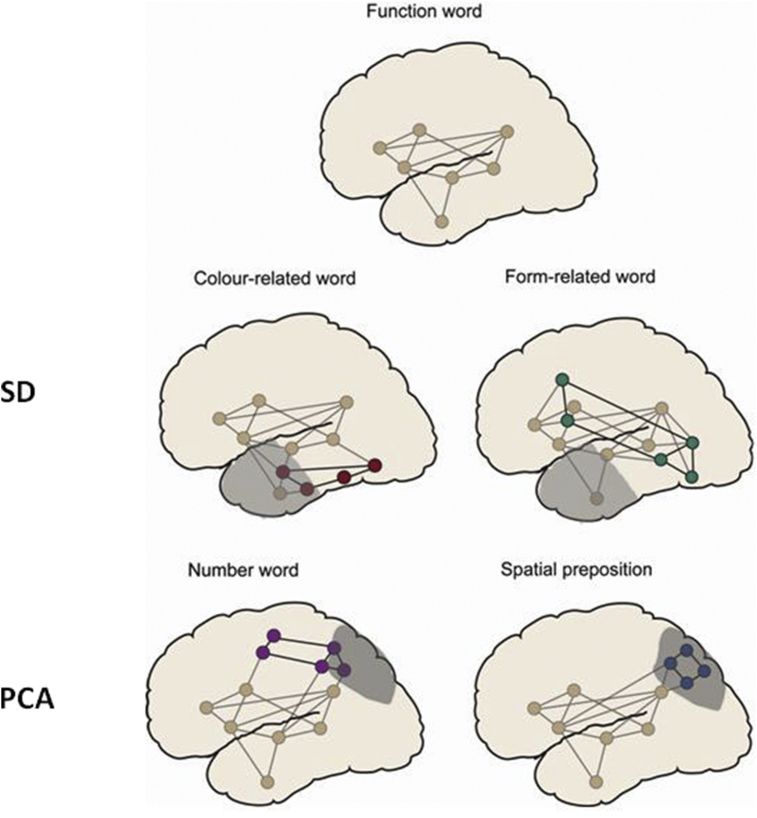
The extended semantic topography model showing semantic circuits for the five word categories used in the present study (Colour, Form, Function, Number and Prepositions). Lesioned areas are highlighted in grey in anterior-temporal regions for SD and in occipito-parietal regions for PCA and cover semantic circuits of word categories predicted to be impaired in each patient group. A set of neurons in the anterior temporal lobe serves as a conceptual ‘hub’ that associates different aspects of semantic knowledge for all categories.

Category-specific activation patterns associated with the meanings of words can best be explained by a proposal of semantic binding circuits that reach into sensory and motor areas of the brain and reflect action- and perception-related features of concepts. In such a proposal, circuits in perisylvian language cortex recruited for processing of any and all words (shown in grey in Fig. 1) are additionally bound to category-specific semantic circuits distributed across additional and different areas (Pulvermüller, 1999, Pulvermüller, 2013). These semantic networks represent information about the objects, properties and/or actions the words are typically used to speak about by members of the language community, and in which their meaning can therefore be ‘grounded’ (see Harnad, 1990). According to this ‘semantic topography’ model, in addition to brain regions recruited by any kind of word stimulus, words referring to colours (e.g., maroon) would receive an extra component of processing from anterior inferior temporal regions (shown in red), whereas words related to object form (e.g., cylinder) would have extra, selective activation in more posterior-temporal regions (shown in green). In the case of SD, since the ventral ‘what’ stream is involved with object identification and recognition, and lesions to this stream lead to deficits specific to object type, SD patients are expected to be more impaired on words carrying visual semantic information about how an object looks (e.g., its colour and form). Specifically, the model predicts that, since SD patients have temporal lobe atrophy, most pronounced in the anterior portion, they would be more impaired on processing colour-related words (with their anterior semantic anchor neurons) than on processing well-matched visually-related words denoting form. In the case of PCA, as patients have atrophy in parieto-occipital regions and since lesions to the dorsal ‘where’ stream lead to deficits in processing spatial concepts and orienting in space (as opposed to deficits in processing and recognising the visual appearance of objects), the model predicts that PCA patients would be more impaired on processing spatial prepositions which describe the relationship of objects in space and, to a lesser degree, number words, since spatial skills are required to perceive how numbers are placed in relation to each other.

### 1.1 SD

SD is marked by a progressive deterioration of all kinds of conceptual knowledge. Patients with SD have impaired comprehension and production affecting the processing of spoken and written words, real objects, pictures, environmental sounds, smells etc. (Bozeat et al., 2000, Coccia et al., 2004, Hodges et al., 1992, Patterson et al., 2007). Despite the comprehensive semantic deficit, the impairment in SD is quite selective in the sense that, apart from semantic memory, most cognitive functions and abilities such as episodic memory, working memory, non-verbal problem solving, visuospatial function and simple calculation skills are relatively well-preserved, at least until the late stages of the disease (Hodges & Patterson, 2007).

In the modern era, SD was first reported by Warrington (1975) who described three patients with an impairment restricted to semantic knowledge. The condition was later given its current name, SD, by Snowden, Goulding, and Neary (1989). With the emergence of brain imaging techniques, it became clear that SD is associated with hypometabolism and atrophy of the anterior inferior temporal lobes (Hodges et al., 1992, Nestor et al., 2006). Though degeneration is bilateral, it is often asymmetrical, particularly in its earlier stages, with atrophy more pronounced on the left in perhaps two-thirds or three-quarters of SD cases (Davies et al., 2004, Hodges et al., 1992, Snowden et al., 1989, Tyrrell et al., 1990). The degree of anterior temporal deterioration in SD, especially in the anterior fusiform gyrus, is profound (Hodges & Patterson, 2007) and correlates positively with the severity of the semantic deficit (Davies et al., 2004, Mion et al., 2010, Mummery et al., 2000, Nestor et al., 2006). The neuropathology in SD is typically abnormal neuronal inclusions of the protein ubiquitin (Davies et al., 2005), or, more specifically, the TAR DNA binding protein (TDP)-43 (Hodges et al., 2010).

### 1.2 PCA

PCA is an atypical form of AD characterised by a progressive and dramatic decline in visuospatial processing. Common symptoms include profound difficulties in visual localisation of objects, right-left disorientation, visual inattention, simultanagnosia and impaired reading/writing due to the visuospatial disorder (Benson et al., 1988, Caine, 2004, Freedman et al., 1991, Mendez et al., 2002, Ross et al., 1996). PCA affects people at a younger age than the more typical amnestic form of AD, with initial symptoms often occurring in the fifth decade. From early in the disease, PCA patients perform poorly on tasks of visual perception (McMonagle, Deering, Berliner, & Kertesz, 2006) and may also have difficulties with object and face recognition (De Renzi, 1986, Rogelet et al., 1996). In contrast to more typical AD, language and memory, especially episodic memory, can be well-preserved or only mildly impaired in PCA (Kas et al., 2011, Mendez et al., 2002).

The term ‘posterior cortical atrophy’ was coined by Benson et al. (1988) who described five patients with a progressive dementia characterised by early onset and “disorders of higher visual function”. PCA affects the posterior regions of the cerebral hemispheres, mainly the parieto-occipital region (Nestor, Caine, Fryer, Clarke, & Hodges, 2003). Atrophy and hypometabolism are bilateral although, as with SD, often asymmetrical. In the case of PCA, it is usually the right side that is more affected (Delazer et al., 2006, Freedman et al., 1991, Nestor et al., 2003). The postmortem diagnosis in PCA is typically AD, though as expected with a different distribution of brain pathology: a higher density of neurofibrillary tangles in posterior regions while anterior regions are less affected (Hof et al., 1997, Tang-Wai et al., 2004). A recent study demonstrated that atrophy remained centred on posterior regions even at the late stage of the disease (Kas et al., 2011).

### 1.3 Semantic word category processing in SD and PCA

SD is characterised by an across-the-board semantic deficit affecting all semantic knowledge (with the exception of simple number knowledge: Cappelletti et al., 2001, Julien et al., 2008). There is, however, some disagreement as to whether all semantic word categories are equally impaired, and the apparent absence of category differences in previous studies of SD may relate to the types of word categories that have been assessed. Most previous studies have focused on the very broad, general categories of (a) nouns versus verbs, (b) names of living things versus artifacts or (c) words referring to concrete versus abstract concepts. The breadth of these categories and the fact that they do not represent homogenous sets of semantic information may render them poorly suited for detecting any fine-grained effects. For example, there are substantial semantic differences between different subtypes of non-living things (e.g., hand-held tools, cooking utensils, large pieces of equipment, musical instruments, buildings etc.). The same holds true for different subtypes of other categories previously studied (e.g., living things, concrete concepts, abstract concepts, verbs, etc.). Comparing such broad categories that are in themselves diverse may be less effective at revealing processing differences than more fine-grained and thus cleaner distinctions.

One study that used word categories defined along more fine-grained semantic distinctions did report differential degrees of impairment in the ability of SD patients to process words from specific semantic subtypes (Pulvermüller et al., 2010). Within the domain of visually-related words, the performance of SD patients in a lexical decision task was more impaired on names of objects with very characteristic colours (such as *grass* or *tomato*) than on words that are typically used to speak about object form. Differences were also found within the domain of action related words; SD patients were more impaired on processing words that refer to actions performed using the face or articulators (e.g., *smile*) than on words that relate to actions performed with the hand (e.g., *pick*). Note that these results are not affected by confounds such as the ones mentioned above related to word frequency, imageability etc., as critical semantic categories and control items had been matched meticulously. The results suggest (i) that the comparison of semantic information at a more fine-grained level may be required in order to reveal any category-specific differences, and (ii) that all categories in SD may not be equally impaired.

With regard to possible category specificity in PCA patients, the more prominent impairments of PCA patients, especially their visuospatial deficits, have understandably received far more attention than their less obvious language and memory deficits. To date, no studies have specifically looked at semantic word processing in patients with PCA, but previously published results suggest that brain regions close to the site of atrophy in PCA may be especially important for the processing of spatial prepositions and number words. For example, an event-related fMRI study investigating the neural circuitry underlying the processing of spatial language (Noordzij et al., 2008) demonstrated the involvement of parietal areas, especially the supramarginal gyrus located in the left inferior parietal lobe, in response to prepositions. In a PET rCBF study, Damasio et al. (2001) also found strong left inferior parietal activation during the naming of spatial relationships using prepositions. Converging evidence comes from a lesion overlap study of patients with impaired knowledge of spatial prepositions (Tranel & Kemmerer, 2004), suggesting a critical role of left inferior parietal regions. As for number words, imaging studies have reported that these also activate parietal regions, especially the intraparietal sulcus (Dehaene, 1996, Pinel et al., 2001) and the right posterior-cingulate cortex (Tschentscher, Hauk, Fischer, & Pulvermüller, 2012), although Tschentscher et al. also found activation in precentral and premotor cortex to number words. Therefore, patients with parietal lobe atrophy might be expected to show relatively selective deficits in the processing of number words and spatial prepositions, with a stronger deficit for the prepositions because number words may also rely on motor regions.

In summary, the main aim of this study is to examine semantic word category processing in SD and PCA patients, looking specifically at possible differences in the degree to which different specific semantic word categories may be impaired in each patient group. Case series of SD and PCA patients performed a test of lexical decision on written words from five different semantic categories: colour (e.g., *yellow*), form (*oval*), number (*seven*), spatial prepositions (*under*), and function words (*also*). The natural language characteristics of these word classes limit the degree to which matching on important variables, especially word frequency, can be accomplished. In particular, number names, spatial prepositions and function words are all very common words that offer essentially no frequency overlap with the other two categories of interest, colour and form words. No matter what the task or type of stimulus used to probe their knowledge, the performance of SD patients in particular is highly sensitive to frequency or familiarity (Jefferies and Lambon Ralph, 2006, Patterson et al., 2006, Woollams et al., 2008). As a result, the category comparisons on which the analyses can focus will be restricted to word types that enabled matching for frequency of occurrence.

### 1.4 Hypotheses

There were three main hypotheses in the present study, based on the following factors: (a) the areas of principal brain atrophy in the two patient groups; (b) previous published results (mainly from functional imaging) suggesting the relevance of these regions to different types of semantic information and word processing; (c) the necessity (noted above) of making comparisons within, rather than across, word-frequency levels; and (d) the fact that SD patients have a general and severe deficit of semantics and language, whereas PCA patients do not. Hypothesis 1, involving factors a, b, and c, was that SD patients would be more impaired on processing words from the colour category than words from the form category (c.f. Pulvermüller & Hauk, 2006). Hypothesis 2, equally deriving from factors a to c, was that PCA patients would be more impaired on processing spatial prepositions than number words. Hypothesis 3, reflecting factor d, was that SD patients would have poorer performance across the board than the PCA cases. Function words not carrying any referential semantic information, and whose neuronal circuits are therefore assumed to be limited to perisylvian language cortex, were included in the study as a control category. Both of the patient groups were expected to perform well with these items because the lesions underlying both syndromes are known to spare perisylvian cortex.

## 2 Methods

### 2.1 Participants

A lexical decision task was administered to 10 patients (6 male) with a clinical diagnosis of SD and 10 patients (5 male) with a clinical diagnosis of PCA. All patients were monolingual native speakers of English, apart from one bilingual SD patient who spoke English as his first language but an additional and early-acquired second language fluently. All patients were recruited from the Department of Neurology at Addenbrooke's Hospital, Cambridge. Table 1 provides demographic information and neuropsychological scores on the Addenbrooke's Cognitive Examination-Revised (ACE-R) and Mini-Mental State Exam (MMSE). Note that, as expected for these disorders, the PCA patients were on average slightly younger than the SD cases. All SD patients were right-handed with an average laterality quotient (L.Q.) of 97.8% (s.d. = 4.8) from a reduced version of the Oldfield handedness inventory (Oldfield, 1971). Eight of the 10 PCA patients were right-handed (mean L.Q. = 93.4%, s.d. = 14.2) while 2 were left-handed (mean L.Q. = −90%, s.d. = 14.1).

**Table 1:** Individual demographic information and neuropsychological test performance for 10 SD patients and 10 PCA patients, ordered for each patient group by overall ACE-R scores as a measure of general cognitive status.

Patient	Age	Years of education	MMSE (30)	ACE-R Total (100)	Attention & orientation (18)	Memory (26)	Fluency (14)	Language (26)	Visuo-spatial (16)
***Semantic Dementia***									
JWi	79	17	25	64	18	12	6	14	14
DB	69	19	25	57	16	10	4	14	13
VV	68	16	25	57	18	11	4	11	13
BC	64	16	25	54	18	8	4	9	15
CR	65	9	25	53	18	9	1	9	16
JH	65	13	23	52	16	11	1	10	14
MS	67	10	26	49	17	4	0	12	16
SM	73	13	22	47	15	5	2	10	15
JWa	63	16	20	41	14	4	0	7	16
DW	64	16	17	37	11	5	3	8	10
Mean	67.7	14.5	23.3	51.1	16.1	7.9	2.5	10.4	14.2
s.d.	5.0	3.2	2.9	8.0	2.3	3.1	2.0	2.4	1.9
***Posterior Cortical Atrophy***									
SM	65	13	23	74	15	18	11	26	4
JB	56	11	23	72	13	18	10	24	7
MMa	62	21	25	71	17	17	6	24	7
RL	52	15	22	70	14	13	8	25	10
MMc	63	11	NT	NT	NT	NT	NT	NT	NT
CS	62	17	NT	NT	NT	NT	NT	NT	NT
JH	50	11	NT	NT	NT	NT	NT	NT	NT
L E	61	13	15	48	10	11	2	19	6
JP	54	14	11	31	5	3	3	19	1
GD	67	18	9	22	4	4	4	8	2
Mean	59.2	14.4	18.3	55.4	11.1	12.0	6.3	20.7	5.3
s.d.	5.8	3.4	6.5	21.8	5.0	6.4	3.5	6.3	3.1

MMSE and ACE-R are reported as whole scores.

ACE-R = Addenbrooke's Cognitive Examination-Revised; MMSE = Mini-Mental State Exam; NT = not tested.

Attention & Orientation, Memory, Fluency, Language and Visuo-spatial are sub-sections of the ACE-R.

With relatively rare conditions like SD and PCA, recruited from a single clinic and within a limited period of time, it is not feasible to do precise matching of the groups for severity. Furthermore, given that these two patient types are principally impaired in different cognitive domains (visuospatial processing in PCA, conceptual knowledge in SD), precise matching for capacity is not really possible or meaningful. The main features characterising the two syndromes were confirmed by ACE scores of the present patient groups, but notably, the mean overall ACE scores in the two groups were actually very close (51.1 *vs* 55.4 out of 100) and not significantly different. In other words, neither group was consistently poorer than the other. We suggest, therefore, that there is no concern about differential severity between the groups. Furthermore, the main conclusions to be drawn will be based on the relative performance on different word classes within rather than between the two patient groups.

Twelve neurologically healthy control participants (7 female) completed the lexical decision task. All were monolingual native speakers of English and were matched to the patients in years of education (mean = 14.5, s.d. = 2.4) and handedness (mean L.Q. = 91.8%, s.d. = 12.6%). Control subjects were also matched in age to the SD patients but were older than the PCA patients (range 61–71, mean = 66.4, s.d. = 3.0). All participants (patients and healthy controls) provided informed, written consent prior to their participation. Ethics approval was obtained from the Local Regional Ethics Committee.

**Fig. 2:**
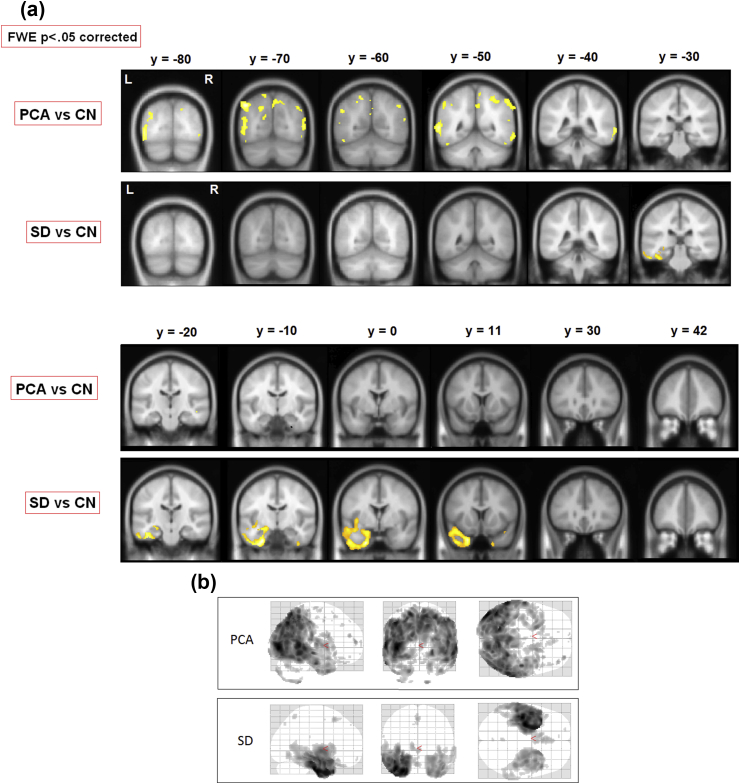
Areas of degeneration in each patient group. (**a**) In a voxel-based morphometry study, PCA patients contrasted with controls (top panel) showed degeneration in posterior temporo-parieto-occipital brain regions while SD patients contrasted with controls (bottom panel) showed anterior temporal lobe degeneration. Family-wise error (FWE) correction was used (*p* < .05); therefore only most extreme areas of degeneration are shown in each patient group. (**b**) Glass brains of the PCA and SD groups showing the same contrasts at a lenient threshold of *p* < .001 (uncorrected).

To assess areas of significant grey-matter degeneration in each patient group, a voxel-based morphometry (VBM) analysis was performed on magnetic resonance imaging (MRI) images acquired on a Siemens Trio 3T system. Healthy comparison data for this analysis were from 19 control participants (age-matched to the SD patients in the current cohort) in a previous study performed on the same scanner. Table 2 provides basic demographic information and scores on basic cognitive tests for this group of control participants. The T1-weighted magnetic resonance (MR) images from the three subject groups were spatially normalised into the same stereotactic space using Statistical Parametric Mapping (SPM) 5 (Ashburner & Friston, 2005). The warped images were then segmented, modulated and finally smoothed using an 8-mm full-width at half-maximum kernel. A *t*-test comparison was performed on the modulated and smoothed grey matter segments. As shown in Fig. 2, significant degeneration in SD was primarily observed in anterior-temporal cortex and was much more pronounced on the left side. In PCA, grey matter density was significantly reduced in posterior parieto-occipital regions of both hemispheres.

**Table 2:** Demographic and basic cognitive test scores for imaging Control participants.

Max Score	MMSE	ACE-R	Attention	Memory	Fluency	Language	Vis-Spat	Sex	Age
	30	100	18	26	14	26	16		
**Control no.**									
1	30	95	18	26	9	26	16	M	65
2	29	95	18	25	13	25	15	M	70
3	30	99	18	26	14	25	16	M	66
4	29	97	18	26	14	25	16	F	65
5	30	97	18	25	12	26	16	F	65
6	30	97	18	26	11	26	16	F	59
7	30	98	18	26	13	26	15	F	62
8	28	93	18	23	13	25	14	M	72
9	30	98	18	26	13	26	15	F	59
10	27	93	18	22	13	24	16	M	70
11	29	93	18	25	9	25	16	M	79
12	29	95	18	26	14	25	12	M	79
13	29	96	18	25	13	25	15	F	66
14	29	89	18	23	9	24	15	M	76
15	29	94	17	25	13	24	16	M	70
16	30	98	18	26	13	25	16	F	63
17	30	95	18	25	12	25	15	F	66
18	29	93	17	23	13	24	16	M	64
19	28	88	18	21	10	23	15	F	61
Mean	29.21	94.89	17.89	24.74	12.16	24.95	15.32		67.21
s.d.	.85	2.96	.32	1.56	1.71	.85	1.00		6.01

It is important to note that the areas of significantly lesioned tissue in our patient populations did not reach into the main perisylvian language areas. Despite substantial atrophy to the anterior temporal lobe in the SD sample, posterior parts of temporal cortex (MNI y coordinate ≤ −30) were not significantly affected; and even in the anterior temporal lobe, the superior temporal gyrus (which is part of the perisylvian region) was relatively spared (see Fig. 2, *y* = −10). In contrast, only quite posterior areas (y coordinate ≤ −50) were significantly affected in our PCA group. The patchiness of significant lesions implies a degree of involvement of the angular gyrus, a region known to contribute to reading and possibly general semantic processing (Binder and Desai, 2011, Geschwind, 1970).

### 2.2 Materials

Lexical stimuli consisted of 120 meaningful words, 24 from each of 5 different lexico-semantic categories: (1) words referring to colours (e.g., *maroon*, *beige*), which are mainly encountered as adjectives (e.g., a *purple* dress) but can also be used as nouns (e.g., *mauve* is a pretty colour; *bronze* is a type of metal); (2) words referring to form or shape (e.g., *circle*, *grid*), which are mainly nouns; (3) prepositions referring to spatial relations (e.g., *under*, *through*); (4) number words (e.g., *twelve*, *million*), which can be used as either adjectives (*twelve* people) or nouns (e.g., *twelve* is an even number); and (5) function words (e.g., *unless*, *also*) including conjunctions, interjections, pronouns and auxiliary verbs. Psycholinguistic variables that are known to affect lexical decision are summarised in Table 3. A different set of healthy native English speaking individuals provided semantic ratings of the words, including arousal, valence, imageability, concreteness and action relatedness, which allowed the lexical stimuli to be further matched for semantic variables. As already indicated in the Introduction, it was not possible to match the words across all five word categories for some properties; in particular, Colour and Form stimuli were lower in word frequency and trigram frequency but rated as more imageable and concrete than Function, Number and Preposition items. However, sub-sets of these word categories—those with strong visual associations (i.e., Colour and Form) and then, separately, the remaining three word categories (Function, Number and Prepositions)—were respectively matched for these variables (see Table 3). The main analyses will be restricted to comparisons of matched subsets.

**Table 3:**
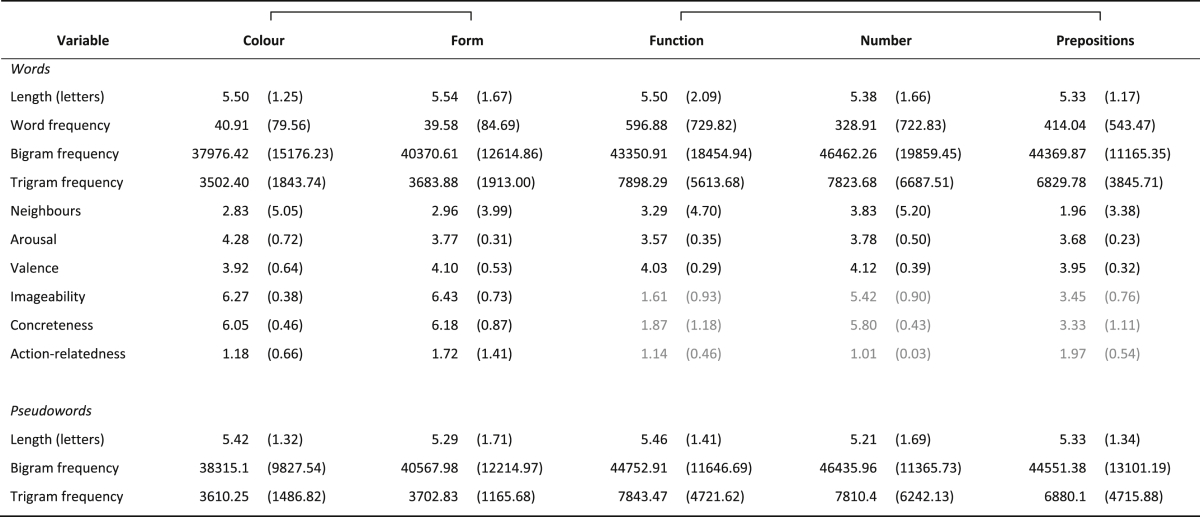
Psycholinguistic and semantic features of the word and pseudoword stimuli.

Mean and s.d. (in brackets) are given for each feature and word category. Frequencies are given in occurrences per million words of standard text. Frequencies (word, bigram and trigram) and neighbours are taken from the CELEX Database. Semantic ratings of arousal, valence, imageability, concreteness and action-relatedness were on 7-point scales with 1 indicating *no relationship* and 7 indicating a *strong relationship.* Brackets at the top indicate that visually related words (Colour and Form) were matched for all the variables and that the remaining three word categories (Function, Number and Prepositions) were matched separately for length, bigram, trigram and word frequency, neighbours, arousal and valence. Differences between matched subsets were n.s. at *p* < 0.05 apart from imageability, concreteness and action-relatedness for the subset that included Function, Number and Prepositions (in grey).

Stimuli prepared for the lexical decision task also included 120 orthographically well-formed pseudowords. Five groups of pseudowords were created so that each group closely matched the words from one of the five meaningful word categories in terms of length and bigram and trigram frequencies (Table 3).

### 2.3 Procedure

Stimuli were presented on a computer screen in large grey letters (point size 20, Arial Bold) against a black background. Participants were seated approximately one metre away from the computer screen. In each trial, participants were presented with either a meaningful word or a meaningless letter string and were instructed to say ‘yes’ if they thought the item was a meaningful standard English word and ‘no’ if they did not think the item was a real word. If they were not sure, they were instructed to guess. A verbal response was adopted on the basis of a previous study in SD (Pulvermüller et al., 2010) in which, during the piloting stage, it became clear that some of the more impaired patients struggled with the dual task of attending to the stimuli and using a button box to indicate a response. Stimulus items remained on screen until a decision was made. As soon as a response was given, the experimenter pressed a button coding the type of response. A fixation cross appeared on the screen for 1 sec between stimulus items. Participants were instructed to keep their gaze fixed on the cross in the middle of the screen and respond to stimuli as quickly and as accurately as possible. After every 80 items, participants were offered a break. Because the experimenter's button presses provide only a delayed (and probably somewhat variable) measure of the participants' actual response times, the data analysis is based on response accuracy as opposed to reaction times.

Clear written and spoken instructions were given to all participants, followed by a practice block to familiarise them with the task. Participants had the opportunity to ask questions and to repeat the practice block if necessary. The experiment was not initiated until participants were comfortable with the task and the experimenter was satisfied that they had understood the instructions.

### 2.4 Statistical analysis

Average hit rates, false positive rates, and *d′* values were calculated for each subject and for each word and pseudoword category. As the ‘visual’ words (Colour and Form) and their respective pseudoword lists were matched for psycholinguistic variables separately, and likewise there was matching across the other three word categories and pseudowords (Function, Number and Prepositions), *d′* values were calculated by using false positive rates collapsed across the sets of pseudowords selected for Colour and Form words, and similarly false positive rates collapsed across the pseudowords for the other three word categories. Average hit rates, false positive rates, and *d′* values were entered into repeated measures analysis of variance (ANOVAs) including the within group variable Word Category and the between group factor Subject Group. Planned comparison analyses were also performed in order to reveal any statistical differences between word categories across and within participant groups.

## 3 Results

Analysis of the performance on the five word categories across the three subject groups revealed significant effects for all three measures: hit rates [*F* (8, 116) = 18.0, *p* < .0001], false positive rates [*F* (8, 116) = 3.3, *p* < .0018] and *d′* values [*F* (8, 116) = 10.1, *p* < .0001]. The SD patients' overall accuracy was considerably poorer compared to controls as revealed by hit rates [87.7% (patients) *vs* 99.9% (controls), *F* (1, 20) = 27.7, *p* < .0001], and *d′* values [2.5 (patients) *vs* 3.9 (controls), *F* (1, 20) = 26.3, *p* < .0001]; false positive rates revealed a marginal effect [19.1% (patients) *vs* 1.5% (controls), *F* (1, 20) = 4.0, *p* = .058]. The overall performance of PCA patients was also reduced relative to controls as measured by hit rates [95.8% (patients) *vs* 99.9% (controls), *F* (1, 20) = 11.6, *p* = .003], false positive rates [8.1% (patients) *vs* 1.5% (controls), *F* (1, 20) = 8.7, *p* = .008] and *d′* values [3.2 (patients) *vs* 3.9 (controls), *F* (1, 20) = 22.5, *p* = .0001].

**Fig. 3:**
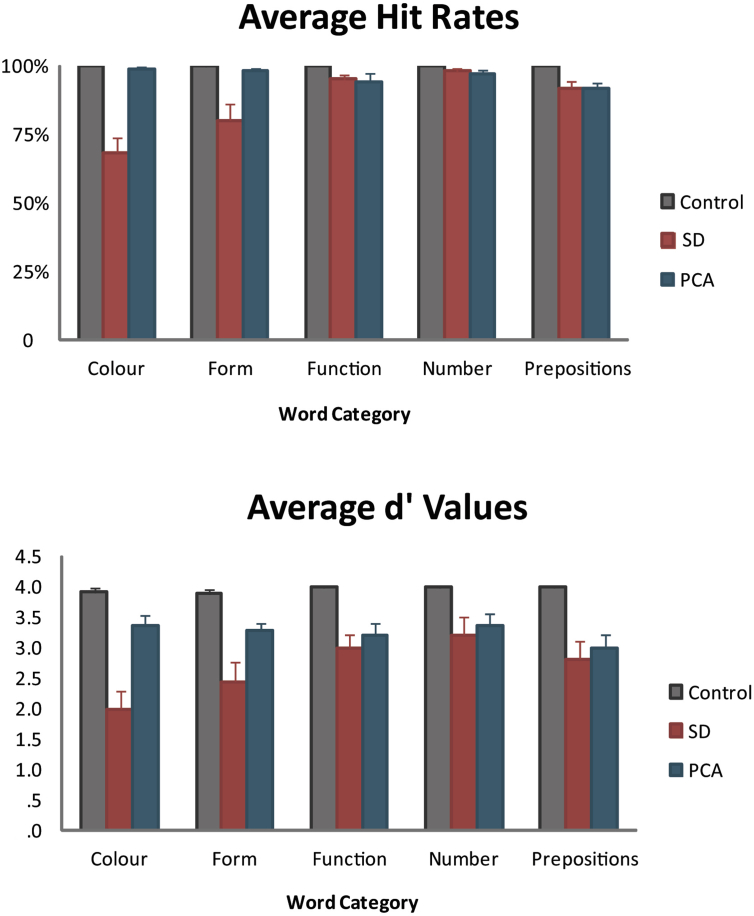
Performance on the lexical decision task. Hit rates and *d′* values with standard error measures are given for SD patients (red bars), PCA patients (blue) and Control subjects (grey) for each of the five word categories: Colour, Form, Function, Number and Prepositions.

There were significant interactions of the Group and Word Category factors in the between-group analysis of both the SD patients versus controls [hit rate: *F* (4, 80) = 20.1, *p* < .0001; false positive rate: *F* (4, 80) = 3.6, *p* = .009; *d′*: *F* (4, 80) = 12.6, *p* < .0001] and in the analysis of PCA patients versus controls [hit rate: *F* (4, 80) = 5.74, *p* = .0004; false positive rate: *F* (4, 80) = 3.1, *p* = .02; *d′*: *F* (4, 80) = 3.4, *p* = .01]. These interactions indicate that the pattern of performance over word types differed between healthy controls and each patient group. Average values for hit rates and *d′* values for the two patient groups and the control group, for each of the five word categories, are presented in Fig. 3.

A 5 × 2 ANOVA with the factors Word Category and Patient Group (SD/PCA) also revealed significant interaction effects on all measures: hit rates [*F* (4, 72) = 18.6, *p* < .0001], false positive rates [*F* (4, 72) = 3.3, *p* < .015] and *d′* values [*F* (4, 72) = 11.3, *p* < .0001], indicating that performance across the five word categories differed significantly between the two patient groups. ANOVAs were then performed separately for each subject group and yielded significant differences amongst word categories in each of the patient groups (for all three measures in SD patients and for hit rates and false positive rates in the PCA group) but no word category differences in the control group.

Planned comparisons of the SD cases versus controls revealed significant differences in each word category, indicating that SD patients were significantly impaired relative to controls for all five of the semantic word categories. The SD patients' poorest performance, however, was on Colour (hit rate = 68.3% *vs* 100%, *p* < .0001; *d′* = 2.0 *vs* 3.9, *p* < .0001) and Form (hit rate = 80% *vs* 99.7%, *p* = .002; *d′* = 2.4 *vs* 3.9, *p* = .0001). In comparing PCA patients with the control group, significant word category differences were also found for each of the five word categories. However, in contrast to the SD group, the PCA patients' best performance was on Colour (hit rate = 98.8% *vs* 100%, *p* = .04; *d′* = 3.3 *vs* 3.9, *p* = .006) while their poorest performance was on Spatial Prepositions (hit rate = 91.3% *vs* 100%, *p* = .0006; *d′* = 3.0 *vs* 4.0, *p* < .0001).

**Fig. 4:**
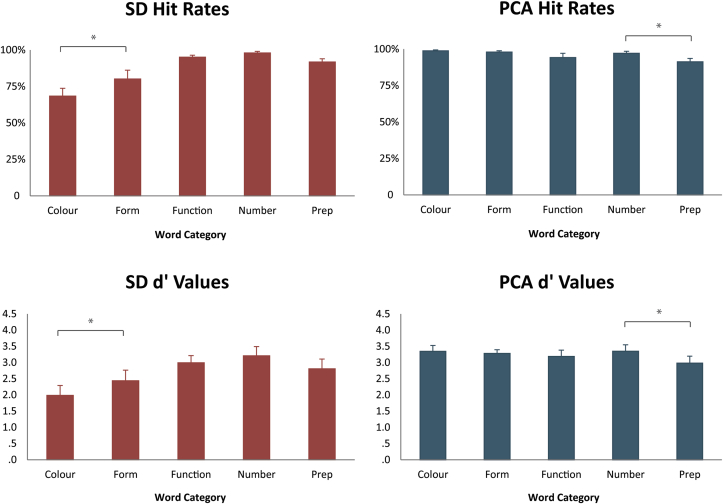
Average hit rates and *d′* values with standard error measures are given for SD patients (left panel) and PCA patients (right panel) for each of the five word categories. Significant differences between matched word categories are highlighted. SD patients performed significantly worse on Colour than on Form as revealed by both hit rates and *d′* values, while the PCA's patients' performance was significantly more reduced for Prepositions than for words from the Number category as revealed by both measures.

Planned comparisons within patient groups between categories matched for psycholinguistic and semantic variables were performed to test hypotheses 1 and 2. As predicted, SD patients performed better on Form than on Colour [hit rate: *F* (1, 9) = 13.7, *p* = .005; *d′*: *F* (1, 9) = 12.6, *p* = .006] and PCA patients better on Number than Prepositions [hit rate: *F* (1, 9) = 17.0, *p* = .0027; *d′*: *F* (1, 9) = 17.8, *p* = .0023]. These word category differences are highlighted in Fig. 4.

Support for Hypothesis 3 is addressed by the lower half of Fig. 3 showing *d′* values. SD patients' ability to discriminate between words and pseudowords was numerically worse than that in the PCA group on all five word categories, even for the three sets of high-frequency words (Prepositions, Number words and Function words). The difference for Colour and Form word processing reached statistical significance [Colour: hit rate = 68.3% (SD) *vs* 98.8% (PCA), *p* < .0001; *d′* = 2.0 *vs* 3.3, *p* = .001; Form: hit rate = 80% *vs* 97.9%, *p* = .01; *d′* = 2.4 *vs* 3.3, *p* = .03].

## 4 Discussion

The aim of this study was to test whether anterior temporal lobe and posterior parieto-occipital lobe regions are of special importance for processing specific semantic word types. Therefore, we examined lexical decisions to words from different lexico-semantic categories in patients with SD or PCA, with the following hypotheses.

The first hypothesis was that SD patients would be more impaired on processing words from the Colour category than from the Form category, because – according to the semantic topography model – the anterior temporal-lobe region that is the primary site of hypometabolism and atrophy in SD is especially relevant to processing of words related to colour knowledge. Planned comparisons between the Colour and Form categories in SD revealed significant differences: SD patients had lower hit rates, higher false positive rates and correspondingly lower d′ values in the Colour than the Form condition. In these two conditions, the words were well matched for lexical frequency, and both the words and pseudowords were well matched for bi-/tri-gram frequencies. Both of these forms of matching are especially important for patients with SD, whose performance in any task is always significantly modulated by both frequency/familiarity and typicality of the stimulus materials (Patterson et al., 2006). The result, therefore, provides strong support for the semantic topography model.

As mentioned previously, a disadvantage for lexical decision to words semantically related to colour knowledge (relative to form-related words) in SD was demonstrated in a previous study (Pulvermüller et al., 2010). The present work, therefore, constitutes a crucial replication of this finding with a different set of SD cases, demonstrating its replicability and reliability, but it also significantly extends the previous work by investigating a novel lexico-semantic word type. The previous work had used words semantically linked to colour knowledge (mostly nouns such as *grass* and *tomato*), whereas our present study employed colour labels that mainly serve as adjectives (e.g., *yellow, beige*), though many can be concrete nouns as well (e.g., *orange, bronze, violet*), and revealed the same category preferential pattern. On the background of linguistic factors that could potentially suggest alternative interpretations, the finding that a predominant colour word deficit is present across lexical category (noun *vs* adjective/noun) and semantic relationship (reference to object characterized by colour *vs* additional direct reference to colour) significantly strengthens the interpretation that the symbols' semantic relationship to colour information is the crucial factor. The observation of a semantic deficit most obvious in the domain of colour knowledge sits well with previous reports about colour processing abnormalities in patients with SD (Rogers et al., 2015, Rogers et al., 2007).

Hypothesis 2, again derived from the semantic topography model, was that PCA patients would be more impaired on processing Spatial Prepositions than Number words. This prediction too was supported by the values for all three measures, and the finding is entirely novel. The prediction was based on the twin facts that (a) the region of primary hypometabolism and atrophy in PCA is parieto-occipital cortex, and (b) that this area is hypothesized to be especially important not only for visuo-spatial processing per se but also for verbal-symbolic knowledge related to this aspect of cognition.

The third prediction, also confirmed by the results, was that SD patients would – across the board – be more impaired at lexical decisions than the PCA cases, although, at the semantic category level, significantly more severe impairments of SD compared with PCA were confirmed for colour and form items, but not for function words, number words and prepositions. This outcome is consistent with the idea of an amodal or multimodal conceptual centre supporting the interactive activation of representations for all semantic categories (Patterson et al., 2007, Rogers et al., 2004). This centre is either contiguous with or partially overlapping the region specifically activated by colour words and possibly important for colour knowledge. Pulvermüller and Hauk (2006) reported maximal category specific activation for colour words in anterior parahippocampal gyrus, centred at MNI coordinate −30 −14 −20, a location included in the area significantly affected in SD patients, which is also part of the left anterior fusiform region of interest where Mion and colleagues reported the best correlation between hypometabolism and general semantic impairments in SD (Mion et al., 2010).

It is worth emphasizing, as Pulvermüller et al. (2010) did, that there is nothing incompatible between the notion of a semantic ‘hub’ (a brain component/region essential for all kinds of knowledge) and the principle of semantic topography (additional regions specific and vital to different kinds of conceptual knowledge/processing). In fact, a recent computational model of semantic learning, which employs a neuronal architecture mimicking area structure and connectivity of fronto-temporal cortex, demonstrates the formation of distributed semantic circuits as a consequence of correlated activity in sensory and motor regions (Garagnani & Pulvermüller, 2016). In this model, and probably likewise in the brain, activity from different sensory and motor modalities can only be linked by way of neuronal pathways passing through multimodal connection hubs. Although the information driving distributed circuit formation may be modality-specific, all types of circuits encompass connector hubs in multimodal areas, therefore explaining the emergence, and necessity, of semantic hubs along with category-specificity (Garagnani & Pulvermüller, 2016).

With respect to the locus of maximum degeneration, in addition to the anterior-posterior dissociation between PCA and SD, there may also be a contribution from laterality. PCA typically results in fairly symmetrical atrophy—as was observed in the present series—or if anything, with a slight bias to right-worse-than-left degeneration (Nestor et al., 2003, Whitwell et al., 2007). SD, in contrast, is characteristically associated with asymmetric temporal lobe atrophy—again as seen in the present series—with left-worse-than-right atrophy being the most frequent pattern. This difference in laterality patterns could, therefore, in theory exert an influence, although we suggest that it is unlikely to be a major factor: despite the commonly asymmetric pattern of atrophy in SD, if one quantifies cerebral glucose metabolism, there is profound and fairly symmetrical hypometabolism in the anterior temporal lobes, even early in the disease (Nestor et al., 2006).

## 5 Conclusion

This study examined the role of different areas of the brain in semantic processing, looking specifically at semantic word category processing in patients with SD and patients with PCA. The two patient groups were selected for the study because their diseases affect specific temporal versus parietal brain regions while leaving cortical language areas largely intact. The main aim of the study was to examine possible differences in the degree to which different lexico-semantic word categories may be impaired in each clinical cohort. Although broad word processing deficits were apparent in both patient groups, the deficit was clearest with colour related words in SD and with spatial prepositions in PCA. These results demonstrate the essential role of word meaning in an apparently ‘non-semantic’ task (lexical decision), and, more importantly, indicate that specific regions outside the perisylvian language cortex are critical for the processing of different semantic categories of words.

SD patients have a severe and general semantic deficit making them impaired at all word categories. Nevertheless, we have demonstrated that – as predicted by the semantic topography model – they have a somewhat greater impairment for words whose meaning-related neuronal circuits ‘reach’ into anterior temporal brain areas affected in SD. PCA patients have no apparent general semantic deficit, as one would expect from the location of their lesion, and so their performance relative to controls is only mildly, though significantly, impaired. Nevertheless, we have demonstrated that – as predicted by the semantic topography model – they have a greater impairment for words whose semantic circuits comprise neurons in parietal cortical areas affected in PCA. What we have learnt from studying these two patient groups is that, at both ends of the spectrum of semantic ability, this kind of approach can highlight small but theoretically telling word-class effects that would otherwise go undetected.

## Figures and Tables

**Table:** 

Colour	Form	Function	Number	Preposition
amber	arc	also	zero	aboard
auburn	circle	although	one	above
azure	cross	anyway	two	across
beige	cube	finally	three	against
bronze	curve	furthermore	four	along
brown	cylinder	hence	five	amid
coral	dot	how	six	among
crimson	ellipse	however	seven	around
emerald	grid	lastly	eight	atop
gold	heart	moreover	nine	behind
green	hexagon	okay	ten	below
grey	octagon	otherwise	eleven	beneath
lilac	oval	shall	twelve	beside
maroon	peak	since	thirteen	between
mauve	plus	still	fifteen	beyond
orchid	point	those	twenty	down
pink	polygon	your	thirty	inside
purple	pyramid	unless	forty	near
scarlet	rectangle	when	fifty	onto
tan	sphere	whether	hundred	out
turquoise	square	whom	thousand	through
violet	star	why	million	under
white	triangle	him	billion	upon
yellow	wedge	yet	trillion	within

## Appendix

List of the lexical stimuli used in the experiment.

## References

[bib1] Ashburner J., Friston K. (2005). Unified segmentation. NeuroImage.

[bib2] Bak T., O'Donovan D., Xuereb J., Boniface S., Hodges J. (2001). Selective impairment of verb processing associated with pathological changes in Brodmann areas 44 and 45 in the Motor Neurone Disease-Dementia-Aphasia syndrome. Brain.

[bib3] Barros-Loscertales A., Gonzalez J., Pulvermüller F., Ventura-Campos N., Bustamante J.C., Costumero V. (2012). Reading salt activates gustatory brain regions: fMRI evidence for semantic grounding in a novel sensory modality. Cerebral Cortex.

[bib4] Benson D.F., Davis R.J., Snyder B.D. (1988). Posterior cortical atrophy. Archives of Neurology.

[bib5] Binder J.R., Desai R.H. (2011). The neurobiology of semantic memory. Trends in Cognitive Sciences.

[bib6] Bird H., Lambon Ralph M.A., Patterson K., Hodges J.R. (2000). The rise and fall of frequency and imageability: Noun and verb production in semantic dementia. Brain and Language.

[bib7] Boulenger V., Mechtouff L., Thobois S., Broussolle E., Jeannerod M., Nazir T.A. (2008). Word processing in Parkinson's disease is impaired for action verbs but not for concrete nouns. Neuropsychologia.

[bib8] Bozeat S., Lambon Ralph M.A., Patterson K., Garrard P., Hodges J.R. (2000). Non-verbal semantic impairment in semantic dementia. Neuropsychologia.

[bib9] Caine D. (2004). Posterior cortical atrophy: A review of the literature. Neurocase.

[bib10] Cappelletti M., Butterworth B., Kopelman M. (2001). Spared numerical abilities in a case of semantic dementia. Neuropsychologia.

[bib11] Coccia M., Bartolini M., Luzzi S., Provinciali L., Lambon Ralph M.A. (2004). Semantic memory is an amodal, dynamic semantic system: Evidence from the interaction of naming and object use in semantic dementia. Cognitive Neuropsychology.

[bib12] Cotelli M., Borroni B., Manenti R., Alberici A., Calabria M., Agosti C. (2006). Action and object naming in frontotemporal dementia, progressive supranuclear palsy and corticobasal degeneration. Neuropsychology.

[bib13] Damasio H., Grabowski T.J., Tranel D., Ponto L.L., Hichwa R.D., Damasio A.R. (2001). Neural correlates of naming actions and of naming spatial relations. NeuroImage.

[bib14] Damasio A.R., Tranel D. (1993). Nouns and verbs are retrieved with differently distributed neural systems. Proceedings of the National Academy of Sciences of the United States of America.

[bib15] Davies R.R., Graham K.S., Xuereb J.H., Williams G.B., Hodges J.R. (2004). The human perirhinal cortex and semantic memory. European Journal of Neuroscience.

[bib16] Davies R.R., Hodges J.R., Kril J.J., Patterson K., Halliday G.M., Xuereb J.H. (2005). The pathological basis of semantic dementia. Brain.

[bib17] De Renzi E. (1986). Slowly progressive visual agnosia or apraxia without dementia. Cortex.

[bib18] Dehaene S. (1996). The organization of brain activations in number comparison: Event-related potentials and the additive-factors method. Journal of Cognitive Neuroscience.

[bib19] Delazer M., Karner E., Zamarian L., Donnemiller E., Benke T. (2006). Number processing in posterior cortical atrophy – a neuropsychological case study. Neuropsychologia.

[bib20] Evans S., Davis M.H. (2015). Hierarchical organization of auditory and motor representations in speech perception: Evidence from searchlight similarity analysis. Cerebral Cortex.

[bib21] Freedman L., Selchen D.H., Black S.E., Kaplan R., Garnett E.S., Nahmias C. (1991). Posterior cortical dementia with alexia: Neurobehavioural, MRI and PET findings. Journal of Neurology, Neurosurgery and Psychiatry.

[bib22] Funnell E., Sheridan J. (1992). Categories of knowledge? Unfamiliar aspects of living and non-living things. Cognitive Neuropsychology.

[bib23] Gainotti G. (2004). A metanalysis of impaired and spared naming for different categories of knowledge in patients with visuo-verbal disconnection. Neuropsychologia.

[bib24] Garagnani M., Pulvermüller F. (2016). Conceptual grounding of language in action and perception: A neurocomputational model of the emergence of category specificity and semantic hubs. The European Journal of Neuroscience.

[bib25] Geschwind N. (1970). The organization of language and the brain. Science.

[bib26] Gonzalez J., Barros-Loscertales A., Pulvermüller F., Meseguer V., Sanjuan A., Belloch V. (2006). Reading cinnamon activates olfactory brain regions. NeuroImage.

[bib27] Grisoni L., Dreyer F.R., Pulvermuller F. (2016). Somatotopic semantic priming and prediction in the motor system. Cerebral Cortex.

[bib28] Harnad S. (1990). The symbol grounding problem. Physica D.

[bib29] Hauk O., Johnsrude I., Pulvermüller F. (2004). Somatotopic representation of action words in the motor and premotor cortex. Neuron.

[bib30] Hauk O., Shtyrov Y., Pulvermüller F. (2008). The time course of action and action-word comprehension in the human brain as revealed by neurophysiology. Journal of Physiology Paris.

[bib31] Hodges J.R., Mitchell J., Dawson K., Spillantini M.G., Xuereb J.H., McMonagle P. (2010). Semantic dementia: Demography, familial factors and survival in a consecutive series of 100 cases. Brain.

[bib32] Hodges J.R., Patterson K. (2007). Semantic dementia: A unique clinicopathological syndrome. The Lancet Neurology.

[bib33] Hodges J.R., Patterson K., Oxbury S., Funnell E. (1992). Semantic dementia: Progressive fluent aphasia with temporal lobe atrophy. Brain.

[bib34] Hof P.R., Vogt B.A., Bouras C., Morrison J.H. (1997). Atypical form of Alzheimer's disease with prominent posterior cortical atrophy: A review of lesion distribution and circuit disconnection in cortical visual pathways. Vision Research.

[bib35] Jefferies E., Lambon Ralph M.A. (2006). Semantic impairment in stroke aphasia vs. semantic dementia: A case-series comparison. Brain.

[bib36] Julien C.L., Thompson J.C., Neary D., Snowden J.S. (2008). Arithmetic knowledge in semantic dementia: Is it invariably preserved?. Neuropsychologia.

[bib37] Kas A., de Souza L.C., Samri D., Bartolomeo P., Lacomblez L., Kalafat M. (2011). Neural correlates of cognitive impairment in posterior cortical atrophy. Brain.

[bib38] Kemmerer D. (2015). Are the motor features of verb meanings represented in the precentral motor cortices? Yes, but within the context of a flexible, multilevel architecture for conceptual knowledge. Psychonomic Bulletin & Review.

[bib39] Kemmerer D., Rudrauf D., Manzel K., Tranel D. (2012). Behavioural patterns and lesion sites associated with impaired processing of lexical and conceptual knowledge of action. Cortex.

[bib40] Kiefer M., Pulvermüller F. (2012). Conceptual representations in mind and brain: Theoretical developments, current evidence and future directions. Cortex.

[bib41] Kiefer M., Sim E.J., Herrnberger B., Grothe J., Hoenig K. (2008). The sound of concepts: Four markers for a link between auditory and conceptual brain systems. The Journal of Neuroscience.

[bib42] Mahon B.Z., Caramazza A. (2008). A critical look at the embodied cognition hypothesis and a new proposal for grounding conceptual content. Journal of Physiology Paris.

[bib43] Martin A., Haxby J.V., Lalonde F.M., Wiggs C.L., Ungerleider L.G. (1995). Discrete cortical regions associated with knowledge of colour and knowledge of action. Science.

[bib44] McMonagle P., Deering F., Berliner Y., Kertesz A. (2006). The cognitive profile of posterior cortical atrophy. Neurology.

[bib45] Mendez M.F., Ghajarania M., Perryman K.M. (2002). Posterior cortical atrophy: Clinical characteristics and differences compared to Alzheimer's disease. Dementia and Geriatric Cognitive Disorders.

[bib46] Mion M., Patterson K., Acosta-Cabronero J., Pengas G., Izquierdo-Garcia D., Hong Y.T. (2010). What the left and right anterior fusiform gyri tell us about semantic memory. Brain.

[bib47] Moscoso Del Prado Martin F., Hauk O., Pulvermüller F. (2006). Category-specificity in the processing of colour-related and form-related words: An ERP study. NeuroImage.

[bib48] Mummery C.J., Patterson K., Price C.J., Ashburner J., Frackowiak R.S., Hodges J.R. (2000). A voxel-based morphometry study of semantic dementia: Relationship between temporal lobe atrophy and semantic memory. Annals of Neurology.

[bib49] Neininger B., Pulvermüller F. (2003). Word-category specific deficits after lesions in the right hemisphere. Neuropsychologia.

[bib50] Nestor P.J., Caine D., Fryer T.D., Clarke J., Hodges J.R. (2003). The topography of metabolic deficits in posterior cortical atrophy (the visual variant of Alzheimer's disease) with FDG-PET. Journal of Neurology, Neurosurgery and Psychiatry.

[bib51] Nestor P.J., Fryer T.D., Hodges J.R. (2006). Declarative memory impairments in Alzheimer's disease and semantic dementia. NeuroImage.

[bib52] Noordzij M.L., Neggers S.F., Ramsey N.F., Postma A. (2008). Neural correlates of locative prepositions. Neuropsychologia.

[bib53] Oldfield R.C. (1971). The assessment and analysis of handedness: The Edinburgh inventory. Neuropsychologia.

[bib54] Patterson K., Lambon Ralph M.A., Jefferies E., Woollams A., Jones R., Hodges J.R. (2006). “Presemantic” cognition in semantic dementia: Six deficits in search of an explanation. Journal of Cognitive Neuroscience.

[bib55] Patterson K., Nestor P.J., Rogers T.T. (2007). Where do you know what you know? The representation of semantic knowledge in the human brain. Nature Reviews Neuroscience.

[bib56] Pinel P., Dehaene S., Riviere D., LeBihan D. (2001). Modulation of parietal activation by semantic distance in a number comparison task. NeuroImage.

[bib57] Pulvermüller F. (1999). Words in the brain's language. Behavioral and Brain Sciences.

[bib58] Pulvermüller F. (2013). How neurons make meaning: Brain mechanisms for embodied and abstract-symbolic semantics. Trends in Cognitive Sciences.

[bib59] Pulvermüller F., Cooper-Pye E., Dine C., Hauk O., Nestor P.J., Patterson K. (2010). The word processing deficit in semantic dementia: All categories are equal but some categories are more equal than others. Journal of Cognitive Neuroscience.

[bib60] Pulvermüller F., Hauk O. (2006). Category-specific conceptual processing of colour and form in left fronto-temporal cortex. Cerebral Cortex.

[bib61] Pulvermüller F., Huss M., Kherif F., Moscoso del Prado Martin F., Hauk O., Shtyrov Y. (2006). Motor cortex maps articulatory features of speech sounds. Proceedings of the National Academy of Science of the United States of America.

[bib62] Rogelet P., Delafosse A., Destee A. (1996). Posterior cortical atrophy: Unusual feature of Alzheimer's disease. Neurocase.

[bib63] Rogers T.T., Graham K.S., Patterson K. (2015). Semantic impairment disrupts perception, memory and naming of secondary but not primary colours. Neuropsychologia.

[bib64] Rogers T., Lambon Ralph M.A., Gerrard P., Bozeat S., McClelland J.L., Hodges J.R. (2004). Structure and deterioration of semantic memory: A neuropsychological and computational investigation. Psychological Review.

[bib65] Rogers T.T., Patterson K., Graham K.S. (2007). Colour knowledge in semantic dementia: It is not all black and white. Neuropsychologia.

[bib66] Ross S.J., Graham N., Stuart-Green L., Prins M., Xuereb J., Patterson K. (1996). Progressive biparietal atrophy: An atypical presentation of Alzheimer's disease. Journal of Neurology, Neurosurgery and Psychiatry.

[bib67] Schnur T.T., Schwartz M.F., Kimberg D.Y., Hirshorn E., Coslett H.B., Thompson-Schill S.L. (2009). Localising interference during naming: Convergent neuroimaging and neuropsychological evidence for the function of Broca's area. Proceedings of the National Academy of Science of the United States of America.

[bib68] Schomers M.R., Kirilina E., Weigand A., Bajbouj M., Pulvermüller F. (2015). Causal influence of articulatory motor cortex on comprehending single spoken words: TMS evidence. Cerebral Cortex.

[bib69] Shtyrov Y., Butorina A., Nikolaeva A., Stroganova T. (2014). Automatic ultrarapid activation and inhibition of cortical motor systems in spoken word comprehension. Proceedings of the National Academy of Science of the United States of America.

[bib70] Shtyrov Y., Hauk O., Pulvermüller F. (2004). Distributed neuronal networks for encoding category-specific semantic information: The mismatch negativity to action words. European Journal of Neuroscience.

[bib71] Simmons W.K., Ramjee V., Beauchamp M.S., McRae K., Martin A., Barsalou L.W. (2007). A common neural substrate for perceiving and knowing about colour. Neuropsychologia.

[bib72] Snowden J.S., Goulding P.J., Neary D. (1989). Semantic dementia: A form of circumscribed cerebral atrophy. Behavioural Neurology.

[bib73] Tang-Wai D.F., Graff-Radford N.R., Boeve B.F., Dickson D.W., Parisi J.E., Crook R. (2004). Clinical, genetic, and neuropathologic characteristics of posterior cortical atrophy. Neurology.

[bib74] Tranel D., Kemmerer D. (2004). Neuroanatomical correlates of locative prepositions. Cognitive Neuropsychology.

[bib75] Tschentscher N., Hauk O., Fischer M.H., Pulvermüller F. (2012). You can count on the motor cortex: Finger counting habits modulate motor cortex activation evoked by numbers. NeuroImage.

[bib76] Tyrrell P.J., Warrington E.K., Frackowiak R.S., Rossor M.N. (1990). Heterogeneity in progressive aphasia due to focal cortical atrophy: A clinical and PET study. Brain.

[bib77] Vigliocco G., Vinson D.P., Druks J., Barber H., Cappa S.F. (2011). Nouns and verbs in the brain: A review of behavioural, electrophysiological, neuropsychological and imaging studies. Neuroscience and Behavioural Reviews.

[bib78] Warrington E.K. (1975). The selective impairment of semantic memory. Quarterly Journal of Experimental Psychology.

[bib79] Warrington E.K., McCarthy R. (1983). Category specific access dysphasia. Brain.

[bib80] Warrington E.K., McCarthy R.A. (1987). Categories of knowledge: Further fractionations and an attempted integration. Brain.

[bib81] Warrington E.K., Shallice T. (1984). Category specific semantic impairments. Brain.

[bib82] Whitwell J.L., Jack C.R., Kantarci K., Weigand S.D., Boeve B.F., Knopman D.S. (2007). Imaging correlates of posterior cortical atrophy. Neurobiology of Aging.

[bib83] Woollams A.M., Cooper-Pye E., Hodges J.R., Patterson K. (2008). Anomia: A doubly typical signature of semantic dementia. Neuropsychologia.

[bib84] de Zubicaray G., Arciuli J., McMahon K. (2013). Putting an “end” to the motor cortex representations of action words. Journal of Cognitive Neuroscience.

